# The association of antipsychotic treatment and side effects with societal recovery and happiness: A naturalistic cohort study of people in long term care for a psychotic disorder

**DOI:** 10.1192/j.eurpsy.2025.891

**Published:** 2025-08-26

**Authors:** S. Crutzen, S. Gangadin, K. H. Hua, W. Veling, S. Castelein

**Affiliations:** 1 Lentis Research, Lentis; 2 Rob Giel Research Center, University Medical Center Groningen, Groningen; 3 Apotheek Spaarne Gasthuis, Spaarne Gasthuis, Haarlem; 4 Psychosis Department, University Medical Center Groningen; 5 Faculty of Behavioural and Social Sciences, University of Groningen, Groningen, Netherlands

## Abstract

**Introduction:**

Antipsychotics are used to manage psychotic symptoms and reduce the risk of relapse. However, the side effects associated with antipsychotic treatment are seen to hinder societal recovery and happiness by antipsychotic users.

**Objectives:**

In this study, we investigate the association of side effects, antipsychotic dose and antipsychotic polypharmacy with societal recovery and happiness.

**Methods:**

Data from a naturalistic longitudinal cohort was used (PHAMOUS, 2013-2021; n> 3000). Mixed effect linear regression models were used to investigate the association between subjective side effect burden, antipsychotic dose and antipsychotic polypharmacy with societal functioning and happiness. Moreover, the association of single antipsychotic side effects with societal recovery and happiness were investigated with mixed effect linear regression models.

**Results:**

The subjective antipsychotic side effect burden and total antipsychotic dose were both negatively associated with societal recovery and happiness. Polypharmacy was additionally negatively related to societal recovery. Cognition, mood and anticholinergic side effects were most strongly associated with societal functioning. Mood, sedation, cognitive, and sexual related side effect were most strongly associated with happiness.

**Conclusions:**

The association between side effects and the antipsychotic dose and societal functioning and happiness in this population in long term care shows the importance of addressing overtreatment at an early stage. Future research should focus on whether addressing side effects, especially mood and cognition related side effects, is beneficial for societal recovery and happiness in the long-term.

**Disclosure of Interest:**

None Declared

